# Research on the optimization method of inventory management of important spare parts of intercity railway

**DOI:** 10.1371/journal.pone.0327852

**Published:** 2025-07-30

**Authors:** Dongyan Wang, Ying Sun, Liang Yu, Kun Shen, Junbo Li, Xia Wu

**Affiliations:** 1 Institute of Computing Technology, China Academy of Railway Sciences Group Co., Ltd., Beijing, China; 2 School of Civil Engineering, Central South University, Changsha, China; Ataturk University, TÜRKIYE

## Abstract

As cities grow, intercity railways are becoming increasingly popular for short trips between neighboring areas. These railways cater well to commuters and travelers, making reliable and cost-effective maintenance crucial. Timely access to spare parts is essential for ensuring the smooth operation of intercity railways. Traditionally, intercity railways lack failure probability data for spare parts, which hampers the support for spare parts ordering decisions, resulting in spare parts management primarily relying on manual experience. This approach often leads to problems like excessive inventory levels and high management costs. To enhance the reliability of intercity railway operations and reduce spare parts management costs, this paper employs the Zebra Optimization Algorithm-Least Squares Support Vector Machine (ZOA-LSSVM) to analyze the reliability of the important Weibull distribution spare parts of the intercity railway and fit the parameters of the reliability function for spare parts. Based on the failure rate, an inventory control model for intercity railway spare parts is established, aiming to minimize total costs while considering constraints such as order point, order quantity, and equipment availability. A genetic algorithm is designed to solve this model. To verify the effectiveness of the model, we select the contact network insulators of Chinese J Intercity Railway as the case study subject. By comparing the fitting performance of several methods, including ZOA-LSSVM, Genetic Algorithm (GA)-LSSVM, LSSVM, and Least Squares Regression (LSR), the effectiveness of ZOA-LSSVM is validated. The experimental results indicate that ZOA-LSSVM can provide better prediction accuracy. Based on this fitting method, spare parts inventory management is conducted. By comparing it with the traditional manual experience method, it is found that the approach proposed in this paper not only ensures the stable operation of intercity railways but also significantly reduces costs by approximately 13.6%. This result fully demonstrates the superiority of the optimization model established in this paper in practical applications and provides new ideas and methods for the management of spare parts for other intercity railways.

## 1. Introduction

The urban population is rapidly growing, accompanied by the continuous expansion of city size and the increasing demand for residents' travel. Intercity railway is one of the priorities for urban development and construction as a mode of transport to improve the convenience of travelling for residents. To ensure that intercity railways can operate systematically and orderly after being put into service, it is essential to prioritize safety assurance [1]. To minimize equipment downtime during maintenance activities, a proactive approach is essential. It involves identifying critical components with complex manufacturing processes and extended lead times. By analyzing equipment wear patterns and component lifespans, these components can be prepurchased and stockpiled in anticipation of future repairs or replacements. Effective spare parts management is crucial for minimizing downtime and maximizing operational efficiency in intercity rail transit. This involves maintaining an optimized inventory level that balances preparedness for both routine maintenance and unexpected repairs [2].

Inventory control primarily focuses on issues such as the timing of orders and the quantity of goods ordered. The basic inventory control strategies can be mainly divided into those considering the order point, namely the (r, S) strategy [3], the (r, Q) strategy [4], and those considering the inspection cycle, including the (T, S) strategy [5] and the (T, Q) strategy [6]. The strategies considering the order point require continuous monitoring of inventory levels. When the inventory level falls below the order point r, the (r, Q) strategy takes the action of ordering Q spare parts, with a fixed order quantity, whereas the (r, S) strategy replenishes the inventory to S, with a variable order quantity. The strategies considering the inspection cycle fix the length of time between two inspections as T. The (T, S) strategy replenishes the inventory to S, and the (T, Q) strategy replenishes with a fixed quantity Q.

Current research on spare parts management commonly builds upon the aforementioned inventory control strategies and introduces further constraints to develop new models. Sun et al. [7] focused on thermal power units in a new power system and significantly reduced spare parts costs by optimizing the (r, Q) strategy. Kabir et al. [8] integrated a replacement strategy with the continuous review inventory strategy and proposed a simulation model to reduce inventory costs. Gan et al. [9] used simulation and genetic algorithms to determine the characteristic values of the proposed model, such as the spare parts order point and maximum inventory, to lower system costs. Feng et al. [10] proposed a solution for the optimization of spare parts allocation in amphibious aircraft, establishing a model with constraints on spare parts availability and quantity, aiming to minimize maintenance costs. Wang et al. [11] based on the (T, S) strategy, established a dynamic optimization mathematical model for optimizing spare parts supply in various scenarios. Guo et al. [12] employed an extended (T, s, S) strategy and developed a dynamic model to optimize inventory control strategies.

For solving inventory problem models, there are primarily two methods. The first commonly used method is simulation. Moustafa et al. [13] proposed a simulation-based Variable Neighborhood Search method to address the joint optimization of maintenance strategies and inventory, based on the (S-1, S) inventory policy. Heraldo et al. [14] introduced a chance-constrained stochastic mixed-integer program and validated the model through simulation to jointly optimize maintenance and spare parts supply. Orlando et al. [15] proposed a Monte Carlo simulation-based method that can provide a good solution for decision-makers in a short time. The advantage of this method is that inventory changes are relatively intuitive. The second commonly used method is intelligent optimization algorithms. Harifi et al. [16] introduced the Emperor Penguin Colony algorithm to address inventory control issues. Keshavarz-Ghorbani and Pasandideh [17] proposed several hybrid metaheuristic algorithms to solve multi-product multi-warehouse inventory models. Vincent et al. [18] established a joint mixed-integer nonlinear programming model for maintenance scheduling and inventory management and used a two-stage genetic algorithm to solve it.

In previous studies, the inventory control of spare parts often did not consider the reliability of the equipment to which the spare parts belong. Reliability refers to the ability of equipment or the entire system to fulfill its function within a specific state and cycle, and it is a guaranteed indicator for the continuous operation of equipment. The continuous operation of equipment necessitates appropriate maintenance strategies, such as preventive and corrective maintenance. The randomness of equipment failures gives rise to the randomness of spare parts demand. Consequently, the varying reliability of equipment directly impacts the diverse demand for maintenance spare parts. Only by comprehensively considering these factors can the optimal strategy for spare parts inventory control be derived. In Kabir’s research, factors such as ordering costs, stockout cycles, holding costs, and replacement costs were considered, and a joint optimization strategy model based on the life of spare parts (t, s, S) was proposed to ensure the minimum unit cost of spare parts [19]. In Semra Tunali’s research, individual spare parts within the system were studied, and a replacement strategy for randomly failing spare parts was proposed. Using statistical principles, spare parts with identical random failure parameters were identified as the subject of study, and a system-wide replacement and continuous inventory inspection strategy was proposed, with simulation methods applied to determine its accuracy [20].

However, when it comes to intercity railway spare parts management, there are several aspects that have not been fully explored. Firstly, there are relatively few studies specifically targeting the inventory control of spare parts for intercity railways, and there are few models that can provide theoretical support for the management of intercity railway spare parts. Secondly, although the inventory management of intercity railway spare parts is influenced by various factors during operation and is closely related to the status of equipment, there are also few practical application analyses of inventory control considering reliability models. Moreover, the reliability requirements for essential spare parts in intercity railways are high, yet their prices are steep and their quantities are limited, resulting in a scarcity of available failure data, which falls into the category of small samples. Currently, there is limited research by scholars on the analysis of small sample failure data concerning the reliability analysis of critical spare parts in intercity railways.

This paper integrates the method of modeling equipment reliability with spare parts control, proposing a Zebra Optimization Algorithm-Least Squares Support Vector Machine (ZOA-LSSVM) method to fit the reliability of critical spare parts that follow a Weibull distribution with small sample sizes. The availability of spare parts is set as a hard indicator, and the objective function is to minimize inventory costs. The prediction of intercity railway equipment reliability is utilized to guide the control of spare parts. An improved Genetic Algorithm (GA) is employed to solve the model, conducting timely and economic analyses of spare parts management to achieve optimization of spare parts inventory management.

The research on spare parts management of intercity railway holds significant theoretical and practical implications. In theory, by combining reliability theory with inventory control strategies, the theoretical framework of spare parts management has been enriched, especially for intercity railways where existing theoretical support is insufficient. Practically, the established inventory control model and optimization methods can effectively reduce the management cost of spare parts for intercity railways, improve the operational efficiency of intercity railways, and ensure the stable operation of the intercity railway system.

The remainder of this paper is organized as follows. Section 2 elaborates on the spare parts management issues for intercity rail transit system. Section 3 applies the LSSVM and ZOA method to spare parts equipment reliability analysis, detailing the working steps. Section 4 proposes a spare parts inventory management model based on Section 3 and solves it using improved GA. Section 5 conducts a case study on spare parts management for the intercity rail transit system using the improved method. And Section 6 summarizes the paper and prospects for future research.

## 2. Problem description

The intercity rail transit system is a complex, multi-disciplinary integrated system [21], with a wide range of equipment specialties including tracks, bridges, tunnels, electrical and signaling, and power supply. Due to the diversity and complexity of these devices, there is a wide variety of rail transit equipment spare parts with multiple models. The spare parts for rail transit equipment have high uncertainty in demand quantity and some parts have low demand, which makes it difficult for managers to fully grasp the consumption patterns and supply timing of spare parts. This leads to numerous challenges in the inventory management of rail transit equipment spare parts. Maintaining a high level of spare parts inventory can tie up a significant amount of a company's working capital and waste management resources, while a low level of spare parts inventory can result in equipment downtime due to a lack of spare parts, leading to excessive downtime losses and affecting operational service levels. Therefore, determining the appropriate spare parts inventory control based on the usage status of rail transit equipment is a critical issue for ensuring the operational safety of intercity railways, improving the efficiency of spare parts inventory management, and reducing inventory costs.

The current maintenance approach for intercity railway components primarily relies on operational hours and existing failures [22]. Maintenance based on operational time may lead to discrepancies between the actual service status of components and the maintenance plan, resulting in situations commonly referred to as “over-maintenance” or “under-maintenance,” which can cause excessive use of spare parts, shortages, or significant stockpiling. In the case of failure-based maintenance, a lack of sufficient spare parts can lead to service disruptions or even major safety incidents. In actual maintenance plans, the cost of spare part consumption is not negligible. This study addresses a key challenge in intercity rail maintenance: balancing the need for spare parts with the associated costs. By incorporating reliability theory and considering failures alongside spare part management expenses, the research aims to achieve several objectives:

- Optimize inventory levels: This ensures sufficient stock for both preventive maintenance and unexpected repairs, minimizing equipment downtime.
- Maximize component utilization: By ensuring spare parts align with actual component usage, we can avoid unnecessary redundancy and optimize the value derived from both components and inventory.
- Reduce inventory costs: Balancing inventory levels with actual needs minimizes unnecessary storage and ordering expenses.

**Fig 1:**
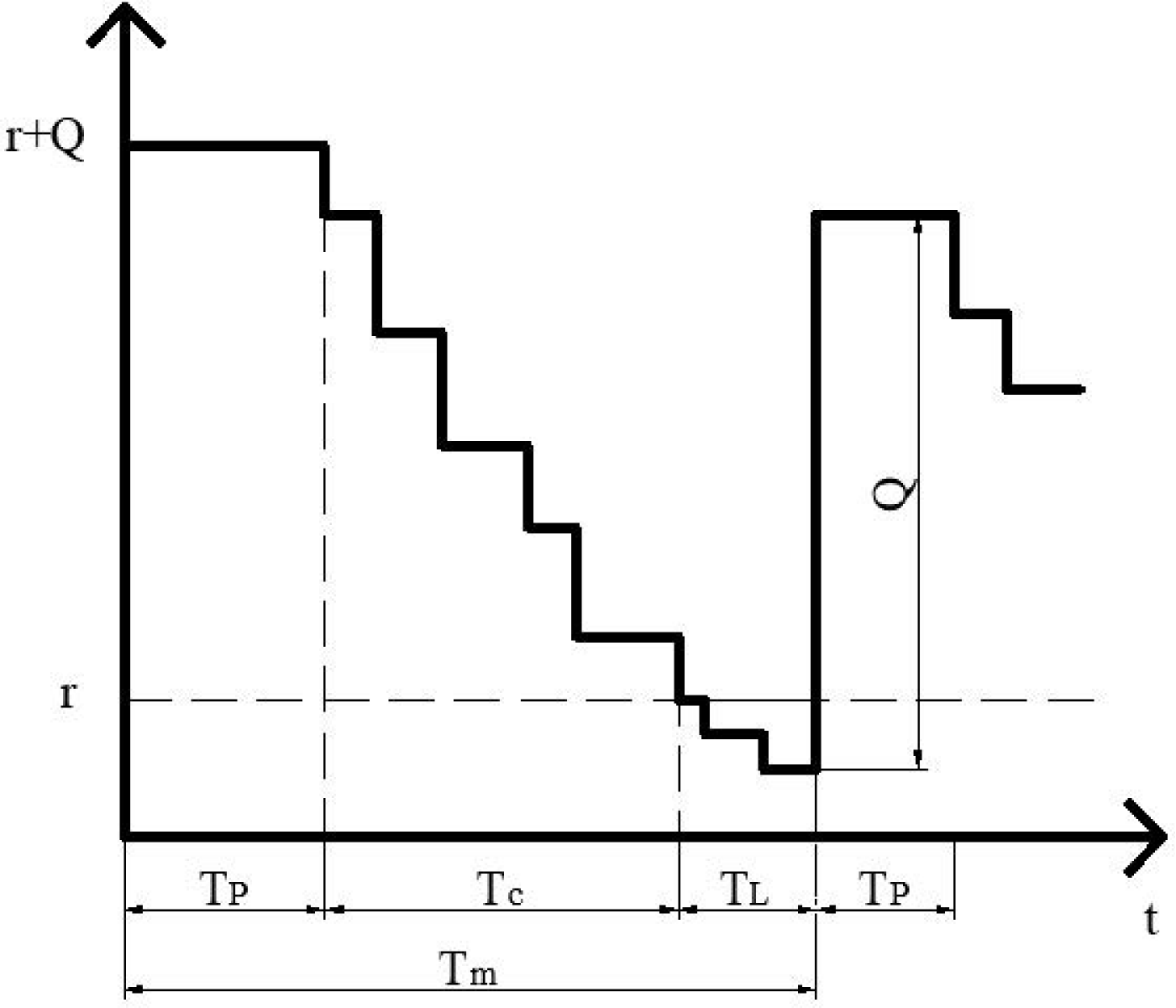
Spare parts inventory changes under the (r, Q) inventory strategy combined with reliability maintenance.

The procurement costs of essential spare parts for intercity railways are high, and historical procurement data show that multiple spare parts need to be purchased at once, which is similar to the literature background employing the (r, Q) strategy. Therefore, this paper integrates the reliability of intercity railway equipment, maintenance strategies, and the commonly used (r, Q) strategy for spare part inventory management for in-depth discussion and research. The changes in the inventory quantity of spare parts are illustrated in Fig 1.

When the quantity of spare parts consumed reaches the order point r, an order is initiated with a quantity of Q. The interval from the initiation of the order to the arrival of the goods, denoted as $T_{L}$ in the figure, is referred to as the lead time for ordering. If no further consumption of spare parts occurs during this period, the quantity of spare parts upon arrival, which is r+Q, represents the maximum inventory level. After the spare parts arrive, preventive maintenance is performed, and the spare parts are stored in the warehouse. During the normal operation of intercity railways, spare parts are not consumed until a failure occurs, at which point they are replaced. The interval during which spare parts are not consumed, represented as $T_{P}$ in the figure, indicates the time from when a failure occurs and spare parts are replaced until only r units of spare parts remain. The time interval between two deliveries of spare parts, denoted as $T_{m}$ in the figure, constitutes a cycle. Since the consumption of spare parts depends on the reliability status of the components, the times depicted in the figure are not necessarily fixed values. Moreover, these times are influenced by the order point r and the order quantity Q. Therefore, this study constructs a model based on r and Q.

## 3. Reliability analysis

Equipment reliability refers to the ability of a device or component to perform its specified functions under specific time or environmental conditions, often used to denote the service life [23]. Various subsystems of intercity railways will experience a range of failures during operation. Understanding the probability of equipment failures is crucial for maintenance. Accurate prediction of equipment failures can, on one hand, provide insights into data such as failure intervals, enabling preventive measures to be taken in advance, reducing downtime, and enhancing equipment availability. On the other hand, failure prediction can be utilized for spare parts procurement and inventory management, effectively avoiding situations where excessive spare parts procurement leads to high inventory costs, or insufficient procurement results in extended maintenance cycles and reduced equipment availability.

### 3.1. The weibull distribution

In the analysis of equipment reliability for intercity railways, physical models or data-driven methods are commonly established as the starting point for evaluation [24]. The physical model analysis method, by thoroughly analyzing the structural characteristics and physical-chemical properties of the object, utilizes collected experimental parameters to assess the reliability or remaining life of the object after a comprehensive analysis of failure mechanisms. However, due to its high cost, this method is typically applied in sophisticated fields. In recent years, data-driven methods have gained widespread use due to their low cost and independence from equipment failure mechanisms. Among them, statistical models such as Weibull distribution, exponential distribution, and normal distribution are common methods for equipment reliability assessment. Since equipment life has a random attribute, and the three-parameter Weibull distribution is well-suited to random data distribution, it is commonly used for research [25]. It mainly includes three parameters: the shape parameter m, the scale parameter k, and the location parameter δ. The shape parameter m primarily influences the shape of the probability density function of the high-speed train axle reducer. The scale parameter k is mainly used to magnify or reduce the scale of the high-speed train axle reducer failure function on the coordinate. The location parameter p only affects the starting position of the probability density function curve on the coordinate axis and does not affect the shape of the probability density function. Since life is often calculated from 0 when analyzing equipment failure rates, in this study, the location parameter δ is set to 0, assuming that the probability density function of intercity railway equipment failures belongs to a two-parameter Weibull distribution.

The failure probability density function of the Weibull distribution can be expressed as:


$$f(t)=\frac{m}{k}{\left(\frac{t}{k}\right)}^{m-1}e^{-{\left(\frac{t}{k}\right)}^{m}}(m,k>0)$$ (1)

The distribution function can be expressed as:


$$F(t)=\int_{0}^{t}f(t)dt=1-e^{-{\left(\frac{t}{k}\right)}^{m}}(m,k>0)$$ (2)

The reliability function can be expressed as:


$$R(t)=1-F(t)=e^{-{\left(\frac{t}{k}\right)}^{m}}(m,k>0)$$ (3)

The failure rate function is


$$\lambda (t)=\frac{f(t)}{R(t)}=\frac{m}{k^{m}}t^{m-1}(m,k>0)$$ (4)

To linearize a two-parameter distribution function, the following transformation can be applied:


$$\ln\left[\ln(\frac{1}{1-F(t)})\right]=m\left[\left(\ln(t)-\ln(k)\right)\right]$$ (5)

If x=ln(t),$y=\ln\left[\ln(\frac{1}{1-F(t)})\right]$, then


y=mx−mln(k) (6)

Historical failure data can be used to determine the values of parameters in the Weibull distribution. Common methods for studying these parameters include the least squares method, maximum likelihood estimation, and support vector machines [26]. Due to the high operational and maintenance requirements of intercity railways in China, the occurrence of faults is relatively low, resulting in fault data for essential spare parts being classified as small sample data. The Least Squares Support Vector Machine (LSSVM) model is well-suited to address the challenges posed by small sample data, while the Zebra Optimization (ZOA) can efficiently optimize the parameters of the LSSVM model. Consequently, a ZOA-LSSVM model is established to achieve the Weibull distribution fitting of the small sample fault data for essential spare parts.

### 3.2. LSSVM

LSSVM is an improved algorithm based on the standard SVM, which is widely applied to problems such as data analysis and pattern recognition. LSSVM transforms the inequality constraints of the standard SVM into equality constraints and applies the least squares method to the optimization of the objective function, converting the quadratic programming problem in SVM into solving a linear equation system in LSSVM, thereby accelerating the convergence speed of iterative processes.

Suppose there are m training samples, where $\left\{(x_{i},y_{i})|i=1,2,...,m\right\},x_{i}\in R^{n}$ represent the input vector of n-dimensional training samples and $y_{i}\in \{1,-1\}$ represent the output group variables. To map the input space to the feature space, a nonlinear function $\varphi (x_{i})$ is employed. The modeling form for estimating the nonlinear function is as follows:


f(x)=b+⟨φ(x),w⟩ (7)

Where b denotes the bias term, ⟨·⟩ refers to the inner product operation, and w represents the weight vector.

Based on the principle of structural risk minimization, the evaluation problem is formulated as an optimization problem:


$$\begin{array}{c} \min J(w,e)=\min(\frac{1}{2}{\left\|w\right\|}^{2}+\frac{1}{2}\gamma \sum_{i=1}^{m}{e_{i}}^{2})\\ s.t.yi=\left\langle w,\varphi (x_{i})\right\rangle +b+e_{i},i=1,2,...,m \end{array}$$ (8)

Where, γ is the regularization parameter used to balance the model complexity and accuracy, and $e_{i}$ represents the error variable.

To address this problem, a corresponding Lagrangian function is constructed:


$$L_{LSSVM}=\frac{1}{2}{\left\|w\right\|}^{2}+\frac{1}{2}\gamma \sum_{i=1}^{m}{e_{i}}^{2}-\sum_{i=1}^{m}\alpha_{i}\left\{\left\langle w,\varphi (x_{i})\right\rangle +b+e_{i}-y_{i}\right\}$$ (9)

Where, $\alpha_{i}$ denotes the Lagrange multiplier corresponding to $x_{i}$.

By taking the derivative of each variable and setting the derivative to zero, we can obtain:


$$\frac{\partial L_{LSSVM}}{\partial w}=0\to w=\sum_{i=1}^{m}\alpha_{i}\varphi (x_{i})$$ (10)

$$\frac{\partial L_{LSSVM}}{\partial b}=0\to \sum_{i=1}^{m}\alpha_{i}=0$$ (11)

$$\frac{\partial L_{LSSVM}}{\partial e_{i}}=0\to \alpha_{i}=\gamma e_{i}$$ (12)

$$\frac{\partial L_{LSSVM}}{\partial \alpha_{i}}=0\to \left\langle w,\varphi (x_{i})\right\rangle +b+e_{i}-y_{i}=0$$ (13)

By eliminating w and $e_{i}$, the four linear problems can be simplified to:


$$\left[\begin{array}{cc} {}*20c0 & E^{T}\\ E & \Omega_{ij}+\gamma^{-1}E \end{array}\right]\left[\begin{array}{c} {}*20cb\\ \alpha \end{array}\right]=\left[\begin{array}{c} {}*20c0\\ y \end{array}\right]$$ (14)

Where, $E={\left[1,...,1\right]}_{m}^{T}$,$y={\left[y_{1},...,y_{m}\right]}^{T}$,$\alpha ={\left[\alpha_{1},...,\alpha_{m}\right]}^{T}$,$\Omega_{ij}=K(x_{i},x_{j})=\varphi (x_{i}{)}^{T}\cdot \varphi (x_{j})$.$K(x_{i},x_{j})$ represents the kernel function. In this study, for computational convenience, the widely adopted Radial Basis Function (RBF) is used.


$$K(x_{i},x_{j})=\exp\left(-\frac{{\left\|x_{i}-x_{j}\right\|}^{2}}{\sigma^{2}}\right)$$ (15)

Where, σ represents the kernel parameter.

Finally, after solving the optimization problem, a linear model for function estimation is obtained.


$$y=\sum_{i=1}^{m}\alpha_{i}K(x_{i},x_{j})+b$$ (16)

The two hyperparameters, γ and σ, are parameters that significantly affect the performance of the LSSVM model and require careful determination.

### 3.3. ZOA

The ZOA, proposed by Trojovská E et al. in 2022, is a novel optimization algorithm that simulates the behavior of zebras to perform optimization tasks. It is characterized by strong optimization capabilities and rapid convergence speed [27]. The ZOA involves several key steps. Initially, a group of zebra individuals is initialized, and random initial solutions are assigned to them. Subsequently, the zebra individuals move according to certain rules, which mimic the behavior of zebras on the savannah. After movement, each zebra updates its solution based on its fitness evaluation. Finally, the algorithm iteratively executes these steps until a stopping criterion is met.

(1) Initialization

The mathematical description of randomly initializing a population in the optimization space is as follows:


$$x_{i,j}=lb_{j}+r\cdot (ub_{j}-lb_{j})$$ (17)

Where, $x_{i,j}$ represents the position of the i th zebra in the j th dimension; $ub_{j}$ denotes the upper boundary of the optimization, $lb_{j}$ denotes the lower boundary of the optimization, and r is a random number within the range [0,1].

(2) Foraging Behavior

In the initial phase, the population members update their positions during the search for food based on the simulation of zebra behavior. Within the ZOA population, the member with the best position is regarded as the pioneer zebra and guides the other population members toward its location in the search space. Consequently, the mathematical description for updating the positions of zebras during the foraging phase can be represented by the following formula.


$$x_{i,j}^{new,P1}=x_{i,j}+r\cdot (PZ_{j}-I\cdot x_{i,j})$$ (18)

$$X_{i}=\left\{ \begin{array}{c} X_{i}^{new,P1},\;F_{i}^{new,P1}<F_{i}\\ X_{i},\;\;\;\;\;\;F_{i}^{new,P1}\geq F_{i} \end{array}\right.$$ (19)

Where, $x_{i,j}^{new,P1}$ represents the new position of the i th zebra in the j th dimension during the first phase; r is a random number within the range [0,1]; PZ denotes the position of the pioneer zebra, which is the best zebra member's position; $PZ_{j}$ represents the j th dimension position of the pioneer zebra; I is the population variation control parameter, with I being a random value from the set {1, 2}; $X_{i}^{new,P1}$ signifies the new position of the i th zebra in the first phase; $X_{i}$ represents the position of the i th zebra; $F_{i}^{new,P1}$ denotes the fitness value of the i th zebra in the first phase; and $F_{i}$ represents the fitness value of the i th zebra.

(3) Predator Defense Strategies

In the second phase, the positions of ZOA population members in the search space are updated by simulating the defense strategies of zebras against predator attacks. Zebras employ different defense strategies depending on the type of predator. In response to lion attacks, zebras defend by fleeing in a zigzag pattern with random lateral turns. Against smaller predators such as hyenas and dogs, zebras become more aggressive, using clustering to confuse and intimidate the predators. In the ZOA design, it is assumed that either of the following two scenarios occurs with equal probability: (i) A lion attacks a zebra, which chooses a fleeing strategy, updating its position near its previous location to evade. (ii) Other predators attack a zebra, which opts for an aggressive strategy, prompting other zebras in the population to update their positions to converge towards the attacked zebra. This can be mathematically described as follows.


$$x_{i,j}^{new,P2}=\left\{ \begin{array}{c} S_{1}:x_{i,j}+R\cdot (2r-1)\cdot (1-\frac{t}{T})\cdot x_{i,j},P_{s}\leq 0.5\\ S_{2}:x_{i,j}+r\cdot (AZ_{j}-I\cdot x_{i,j}),P_{s}>0.5 \end{array}\right.$$ (20)

$$X_{i}=\left\{ \begin{array}{c} X_{i}^{new,P2},F_{i}^{new,P2}<F_{i},\\ X_{i},F_{i}^{new,P2}\geq F_{i}, \end{array}\right.$$ (21)

In the formula, $x_{i,j}^{new,P2}$ represents the new position of the i th zebra in the j th dimension during the second phase; R is a constant value of 0.01; $P_{s}$ denotes the switching probability for choosing either a fleeing or an aggressive strategy; t and T represents the current iteration and the maximum number of iterations in the ZOA, respectively; AZ signifies the position of the zebra under attack; $AZ_{j}$ represents the j th dimension position of the zebra under attack; $X_{i}^{new,P2}$ indicates the new position of the i th zebra in the second phase; and $F_{i}^{new,P2}$ denotes the fitness value of the i th zebra in the second phase.

### 3.4. Construction of the ZOA-LSSVM model

In the LSSVM model, the parameters γ and σ significantly influence the regression performance of the model. Therefore, this paper employs the ZOA to optimize these parameters, aiming to enhance the model's accuracy. The specific modeling process of the ZOA-LSSVM model is as follows:

Step 1: Normalize the fault samples of the overall fault data for preprocessing, using the following formula:


$$x_{i}=\frac{x_{i}-x_{i\min}}{x_{i\max}-x_{i\min}}$$ (22)

Step 2: Initial Parameter Setting: Set the initial values for four parameters during the execution: the size of the zebra population, the maximum number of iterations $T_{\max}$, and the upper and lower bounds of the zebra population positions.

Step 3: Initialize Population Positions: Initialize the positions of the zebra population and set the current iteration number t to 1.

Step 4: Define Fitness Function: The fitness function is defined as the mean squared error between the predicted and actual values. Calculate the fitness for each individual and select the individual with the current best fitness. Set the position of this individual as the current optimal position.


$$f=\frac{1}{\sqrt{\frac{1}{n}{\sum_{k=1}^{m}\left(y_{k}-y_{k}^{\prime }\right)}^{2}}}$$ (23)

Step 5: If $t<T_{\max}$, update I, r, and $P_{s}$.

Step 6: Update the zebra positions according to the formula based on the value of $P_{s}$.

Step 7: After each iteration, recalculate the fitness and compare it. Identify and update to the optimal position. If the termination conditions are met, output the optimal individual position; otherwise, return to Step 5 and continue the execution until the optimal values of γ and σ are obtained.

Step 8: Substitute the optimized γ and σ into the LSSVM model to obtain the fitting parameters of the Weibull model.

## 4. Inventory control model

### 4.1. Model assumptions

Given the complexity of the actual conditions of intercity railway components, and focusing on the issues of primary concern in this study, the following assumptions are proposed:

(1) Intercity railway spare parts are inspected periodically. Upon detection of a failure, the failed part is immediately replaced with a new one, and the failed component is discarded without further repair.
(2) The operation of intercity railways is assumed to be in a normal state; hence the reliability of spare parts follows the laws of the reliability function.
(3) The reliability of spare parts is assumed to be 1 upon initial use. After replacement, the number of spare parts remains constant, and the reliability of the newly replaced parts is also 1.
(4) The reliability function is identical for all spare parts of the same type.
(5) The states of different spare parts are assumed to be independent of each other, with no mutual influence.

### 4.2. Objective function

The costs associated with intercity railway spare parts primarily include procurement costs, ordering costs, storage costs, shortage costs due to stockouts, and consumption costs. Procurement costs are fixed within each ordering cycle. Ordering costs are proportional to the batch size and quantity of spare parts ordered. Storage costs are related to the quantity of spare parts held and the duration of their holding. Shortage costs refer to the expenses incurred when the supply of inventory spare parts is interrupted, preventing the normal operation of intercity railways, and are proportional to the duration of the shortage. Consumption costs are the expenses generated during the maintenance of intercity railways due to the consumption of spare parts. In this study, these include the costs of preventive maintenance and corrective maintenance for spare parts, which are related to the reliability and failures of the components. Therefore, the objective function, aiming to minimize costs, can be expressed as follows:


$$\min C_{T}=C_{F}+Qc_{I}+c_{S}\times I_{m}\times T_{m}+C_{P}+Qc_{D}+c_{u}\times T_{u}$$ (24)

In the formula, $C_{T}$ represents the total cost, $C_{F}$ represents the procurement costs, Q represents the number of spare parts purchased each time, $c_{I}$ represents the price per spare part, $c_{S}$ represents the storage cost per unit of time for spare parts, $I_{m}$ represents the average inventory level within an ordering cycle, $T_{m}$ represents the duration of an ordering cycle, $C_{P}$ represents the cost of preventive maintenance, $c_{D}$ represents the cost of replacing a single spare part during corrective maintenance, $c_{u}$ represents the cost of stockouts per unit of time, and $T_{u}$ represents the duration of the stockout.

(1) Average Inventory Level

The inventory level is denoted by I, and the average inventory quantity $I_{m}$ within a cycle is given by the following formula:


$$I_{m}=\frac{\int_{0}^{T_{m}}I(t)dt}{T_{m}}$$ (25)

Where, I(t) represents the inventory level at time t._._ According to the research by Gao [28], I(t) can be expressed as follows:


$$I_{t}=\max(Q+r-N\lambda T_{L}-N\lambda t,0)$$ (26)

Where r represents the reorder point inventory, N represents the number of components, λ is the failure rate, and $T_{L}$ represents the lead time for ordering.

(2) Ordering Cycle

The duration of an ordering cycle consists of the time for preventive maintenance and other non-consumption periods of spare parts, the total time for repair until the spare parts are consumed to the reorder point, and the lead time for ordering, as shown in the following formula:


$$T_{m}=T_{P}+T_{C}+T_{L}$$ (27)

Where, $T_{P}$ represents the sum of the preventive maintenance time ${T_{P}}^{\prime }$ and the time $T_{PN}$ from normal operation until a failure occurs, while $T_{C}$ represents the total time consumed by spare parts until reaching the reorder point.

According to Diallo [29], the time $T_{C}$ consumed for replacing a spare part during a repair is the mean value of an exponential distribution, as shown in the following formula:


$$T_{C}=-\int_{0}^{\infty }uxe^{-ux}dx=\frac{1}{u}$$ (28)

(3) Stockout Time

The states of N components are mutually independent, thus the probability of experiencing m component failures within the lead time for orders can be obtained through the binomial distribution, as shown in the following formula:


$$P_{m}=\left(\begin{array}{c} {}*20cm\\ N \end{array}\right)\lambda {T_{L}}^{m}(1-\lambda T_{L}{)}^{N-m}$$ (29)

During the lead time for ordering, when the number of spare parts required for replacement due to failures exceeds the order point, and the spare parts have not yet arrived, this can lead to a shortage of spare parts, causing intercity railways to be unable to operate normally. The downtime can be expressed as follows:


$$T_{a}=\{\begin{array}{cc} {}*20c0 & m<r+1\\ \sum {\mathrm{\backslash nolimits}}_{r+1}^{i}\frac{m+1-(r+1)}{m+1}T_{L} & m\geq r+1 \end{array}$$ (30)

Consequently, within a single ordering cycle, the duration of spare parts stockout can be denoted as follows:


$$T_{u}=\sum {\mathrm{\backslash nolimits}}_{m=0}^{N}P_{m}T_{a}$$ (31)

### 4.3. Constraints

This study employs the (r, Q) strategy for inventory control of spare parts in intercity railways. Considering the high safety and reliability requirements during the operation of intercity railways, the main constraints include the ordering cycle, order point, order quantity, and availability.

(1) Ordering Cycle

An ordering cycle encompasses the time for preventive maintenance, the time to consume spare parts to the order point, and the lead time for ordering. Taking into account the special case where the initial inventory of spare parts at the beginning of a cycle equals the order point, the ordering cycle should not be shorter than the lead time for ordering.


$$T_{m}\geq T_{L}$$ (32)

(2) Order Point

The order point must be non-negative, and thus can be represented by the following formula:


r≥0 (33)

(3) Order Quantity

The number of spare parts ordered within a cycle must not be less than the number of failures that occur, hence it can be represented by the following formula:


$$Q\geq N\lambda T_{m}$$ (34)

(4) Availability

According to the research by Gao [28], this study represents availability as the ratio of component operating time to the duration of a cycle within a cycle. Since the operating time can be expressed as the total cycle duration minus the downtime, which includes preventive maintenance time, failure repair time, and downtime due to spare part shortages, availability can be set to a minimum value based on the actual needs of the managers. Therefore, availability is represented by the following formula:


$$A=1-\frac{T_{P}+QT_{c}+T_{u}}{T_{m}}\geq B$$ (35)

Where, A represents availability, and B represents the minimum availability.

### 4.4. Customized Genetic Algorithms

Given the strong adaptability and global search capabilities of Genetic Algorithms, which do not require conditions such as continuity and differentiability in the solution model, and the inherent parallel optimization method using probability theory, GAs can accurately and quickly find the optimal solution. This study employs Genetic Algorithms to solve the aforementioned model. The solution approach involves encoding the two decision variables, order point, and order quantity, to generate several initial populations. Then, based on the biological evolutionary process, continuous iterations are performed to ultimately obtain an approximate optimal solution.

The specific process is as follows.

(1) Encoding and Decoding

**Fig 2:**
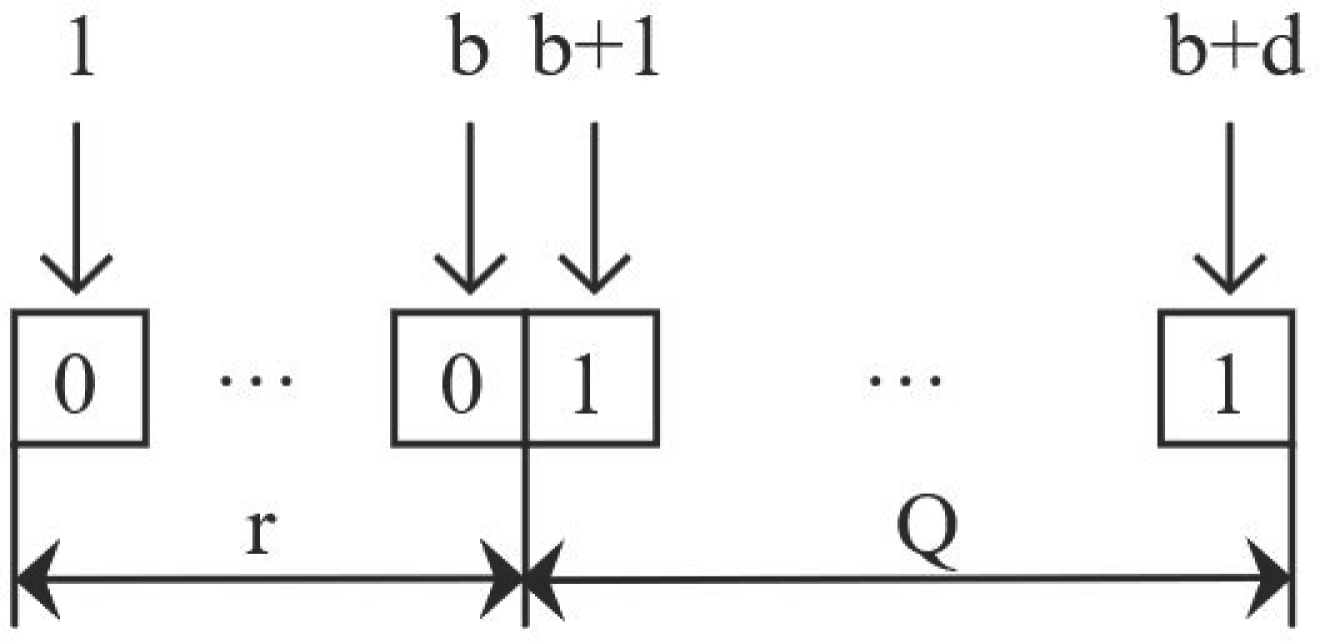
Coding schematic.

There are three commonly used encoding methods. Considering the model established in this study requires determining the order point r and order quantityQ, a binary encoding method that is convenient and straightforward is chosen. The precision is set to 0.1, with the minimum order point and minimum order quantity being 0. The historical maximum order point is $r_{m}$, and the maximum order quantity is $Q_{m}$. Given $2^{a}<10r<2^{b}$ and $2^{c}<10Q<2^{d}$, the encoding length isb+d. The encoding diagram is shown in Fig 2. The two segments of the gene correspond to the numbers of the order point and order quantity, respectively, and the chromosome corresponds to the inventory control scheme.

Decoding involves converting the binary strings representing the order point and order quantity back into decimal numbers.

For the order point, the decoding formula is:


$$r=\frac{r_{m}}{2^{b}-1}\cdot \sum_{i=1}^{b}A_{i}2^{i-1}$$ (36)

For the order quantity, the decoding formula is:


$$Q=\frac{Q_{m}}{2^{d}-1}\cdot \sum_{i=1}^{d}A_{i}2^{i-1}$$ (37)

(2) Chaotic Map Initialization of the Population

Experiments have proven that using chaotic maps to generate random numbers significantly improves the fitness function values. Replacing conventional uniformly distributed random number generators with chaotic maps can yield better results, especially when the search space contains many local solutions, making it easier to find the global optimum. The use of chaotic sequences for population initialization, selection, crossover, and mutation operations affects the entire process of the algorithm and often achieves better results than pseudo-random numbers [30]. Therefore, this study uses the commonly used logistic map for population initialization. The expression for the chaotic map is as follows:


$$x_{i+1}=ax_{i}(1-x_{i})$$ (38)

Where, a is the control parameter, a∈[0,4]

(3) Fitness Function

In this study, the lower the total cost of an individual in the population, the better the individual is considered. Conversely, the higher the fitness function value, the better the individual is deemed. The fitness function is directly set as the negative of the objective function for spare parts management.


$$f=-\min C_{T}$$ (39)

(4) Selection

The tournament selection strategy is employed. At each iteration, two chromosome individuals (with replacement) are drawn from the population, and the one with the best fitness value is selected to enter the offspring population. This process is repeated until the size of the new population reaches that of the original population.

(5) Crossover

Crossover is performed to generate new individuals. Considering the use of binary encoding, this study selects the efficient and available single-point crossover algorithm. Specifically, a crossover rate of 0.8 is set [31]. A random probability value between 0 and 1 is generated, and if this random probability exceeds the set crossover rate, the parents undergo crossover at a random point.

(6) Mutation

Under the condition of binary encoding, the commonly used basic bit mutation operation is employed. Generally, the mutation probability is not high, so a mutation rate of 0.01 is selected [31]. A random probability is generated, and if this random probability exceeds the set mutation rate, a mutation position is randomly chosen using a random number, and the encoding at that position is mutated. In this study, which uses 0–1 encoding, the mutation involves flipping between 0 and 1.

(7) Termination Condition

The maximum number of iterations is set for the population to undergo 100 rounds of selection, crossover, and mutation operations. Once the operations reach 100 times, the best solution is returned, and no further crossover, mutation, or other operations are conducted.

### 4.5. Critical spare parts failure prediction and inventory management process

**Fig 3:**
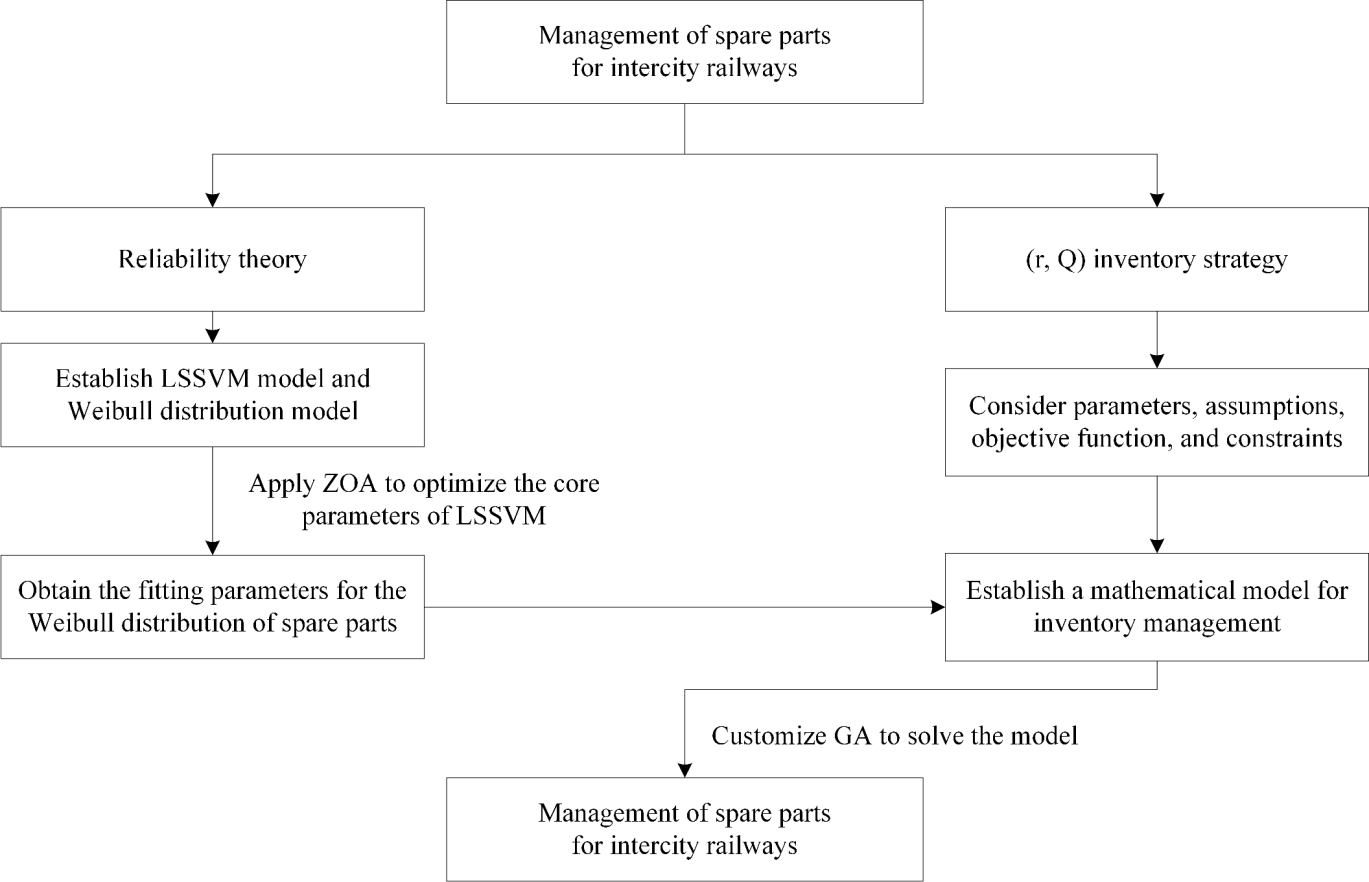
Spare parts failure prediction and inventory management process.

First, the fault data is normalized, and then the dataset is input into the LSSVM model. The ZOA algorithm is employed to optimize the core parameters of the LSSVM, in conjunction with the Weibull model, to obtain the optimal fitting parameters. Based on the (r, Q) strategy and the failure distribution, the assessment of spare part shortages and remaining probabilities during the lead time is performed. An objective function, variables, and constraints are set to construct the inventory control model, and a genetic algorithm is customized for solving the model. This study presents the management approach for essential spare parts in intercity railways as illustrated in Fig 3.

## 5. Case study

We take the suspension composite insulator of the traction power supply system in the Chinese J Intercity Railway as an example to analyze the model proposed in this study. In the intercity railway, approximately every 60 meters along one side of the track, a contact network is set up. Randomly selecting the contact network within a 1 km range, N is set to 16. Considering the importance of components, the availability is set to 0.99. Relevant parameters of the insulator were obtained through consulting experts and reviewing literature [32]. Due to confidentiality requirements, some data have been scaled, but this does not affect the validation of the method in this paper. The data used in this case are shown in Table 1.

**Table 1:** Relevant Parameters.

Notation	Value
N /Pcs	16
$c_{I}$ /Yuan	10000
$C_{F}$ /Yuan	1000
$c_{s}$ /Yuan	500
$c_{u}$ /Yuan	2000
$T_{L}$ /d	30
$T_{C}$ /d	0.2
${T_{P}}^{\prime }$ /d	0.2

### 5.1. Reliability model parameter analysis

**Fig 4:**
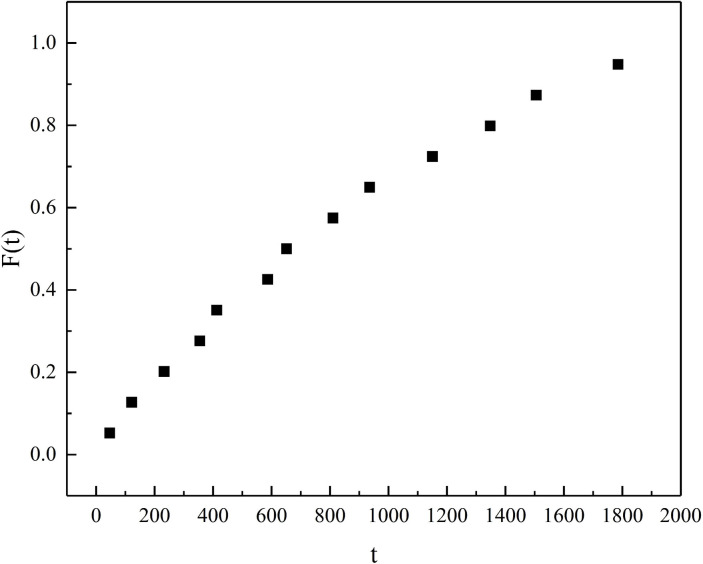
Weibull probability diagram.


The insulator failure data are obtained based on research at J Intercity Railway and sorted by time. The median rank is calculated using (40). With equipment failure time as the horizontal axis and median rank as the vertical axis, a Weibull probability plot can be obtained, as shown in Fig 4. From the graph, it can be observed that the data points are distributed around a straight line, indicating that the sample likely follows a Weibull distribution [33].

$$F(t)=\frac{i-0.3}{n+0.4}$$ (40)

Where, F(t) represents the median rank, i is the sequence number of the current data, and *n* is the total amount of data.

**Fig 5:**
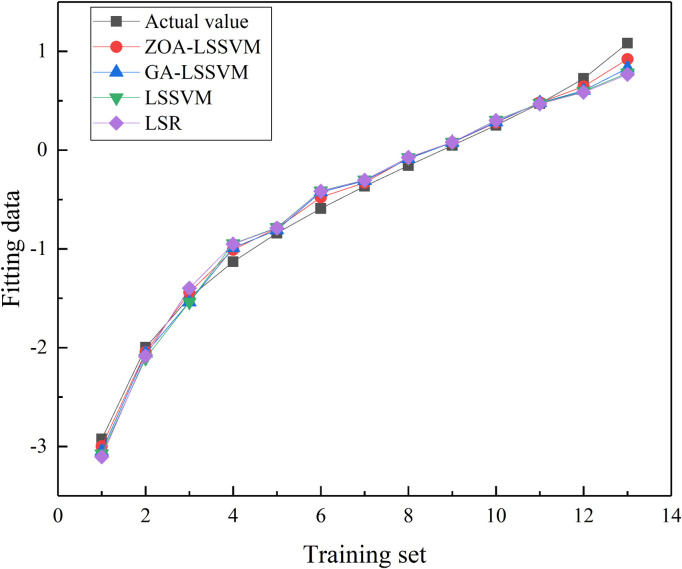
Fitting results.

**Fig 6:**
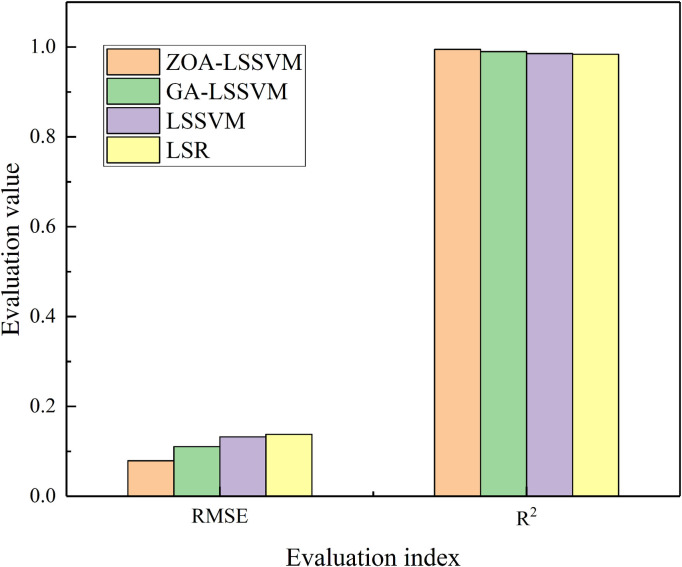
Comparison of model prediction performance metrics.

These data are processed according to Equation (6) and divided into two groups: data 1–10 are used as the training set, and data 11–13 are used as the test set for validation analysis. To verify the effectiveness and accuracy of the ZOA-LSSVM algorithm, comparisons are made with GA-LSSVM, LSSVM, and Least Squares Regression (LSR). The commonly used coefficient of determination ($R^{2}$) and root mean square error (RMSE) are selected for quantitative evaluation. The sample data and fitting results are shown in Table 2, the fitting results are illustrated in Fig 5, and the error analysis is presented in Fig 6.

**Table 2:** Failure Samples and Fitting Results.

Index	Processed data		Fitting results			
	x_i_	y_i_	ZOA-LSSVM	GA-LSSVM	LSSVM	LSR
1	0	−2.9252	−2.9971	−3.0499	−3.0747	−3.1058
2	0.2633	−1.9975	−2.0442	−2.0566	−2.1067	−2.0858
3	0.4401	−1.4916	−1.4400	−1.5379	−1.5379	−1.3981
4	0.5563	−1.1297	−1.0081	−0.9890	−0.9472	−0.9509
5	0.5980	−0.8394	−0.7895	−0.8092	−0.7831	−0.7894
6	0.6946	−0.5905	−0.4775	−0.4243	−0.4127	−0.4152
7	0.7232	−0.3665	−0.3288	−0.3105	−0.3022	−0.3044
8	0.7831	−0.1569	−0.0821	−0.0871	−0.0741	−0.0726
9	0.8225	0.0465	0.0762	0.0786	0.0796	0.0802
10	0.8793	0.2522	0.2790	0.2850	0.3030	0.3000
11	0.9228	0.4712	0.4828	0.4781	0.4743	0.4687
12	0.9531	0.7249	0.6447	0.6031	0.5949	0.5861
13	1	1.0824	0.9210	0.8288	0.7812	0.7674

It can be seen that the coefficient of determination R2 under the ZOA-LSSVM algorithm is relatively high, while the RMSE is relatively low. This indicates that the ZOA-LSSVM algorithm can provide reliable parameter estimates for the reliability of intercity railway contact network insulators under small sample conditions, and the estimation accuracy has been improved. The reliability model parameters obtained are m = 167.7904 and k = 5.0636.

For small sample sizes, the commonly used Kolmogorov-Smirnov (K-S) test method [34] can be employed to assess the goodness-of-fit of the model. The observed cumulative distribution function is denoted as $F_{i}$, and the expected cumulative distribution function calculated from the fitted model is also ${F_{i}}^{\prime }$. Let $D_{i}=\left|F_{i}-{F_{i}}^{\prime }\right|$ be the test statistic for the K-S test. With a sample size of 13 and a confidence level set at 0.05, the critical value $D_{c}$ is found to be 0.361. The calculated value D is 0.2486. Since $D<D_{c}$ it is concluded that the Weibull model is appropriate.

### 5.2. Order cost analysis

**Fig 7:**
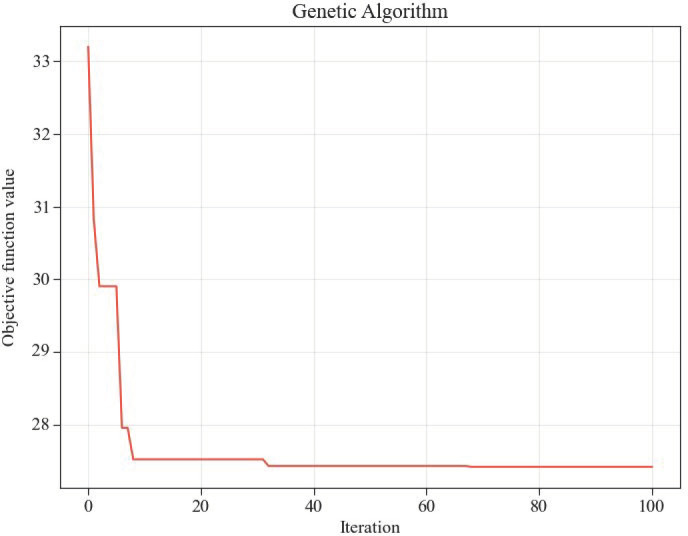
Objective convergence.

The relevant parameter settings for the genetic algorithm primarily include a population size of 100, a crossover probability set at 0.8, a mutation probability set at 0.01, and the termination condition set to reach a maximum number of iterations, which is 100. After conducting 30 experiments, the genetic algorithm converges around the 30th generation. A typical convergence graph is shown in Fig 7.

The final optimal order point is determined to be 1, with an order quantity of 3, availability of 0.9993, and a total cost of 276,520 yuan. The results of this model are compared with historical empirical procurement data, as shown in Table 3.

**Table 3:** Comparison between the model proposed and the actual procurement situation.

Method	Artificial empirical method	The proposed model
Order point	1	1
Order Quantity	1-10	3
Availability	cannot guarantee	0.9993
Total cost/yuan	320,000	276,5200

As evidenced by the actual procurement of insulators for Chinese J Intercity Railway and the comparison with the results obtained from the model in this paper, the order quantity determined by the manual experience method is uncertain. It may lead to insufficient order quantities causing stockouts and operational disruptions, or it may result in excessive orders increasing inventory holding costs. Moreover, the manual experience method cannot guarantee the normal operating time of the insulators, which is the availability proposed by the model constructed in this study, potentially leading to significant safety incidents. The model proposed in this study significantly meets the availability of the insulators and also reduces inventory redundancy, lowering the total cost by approximately 13.6%.

### 5.3. Analysis of equipment availability relationships

**Fig 8:**
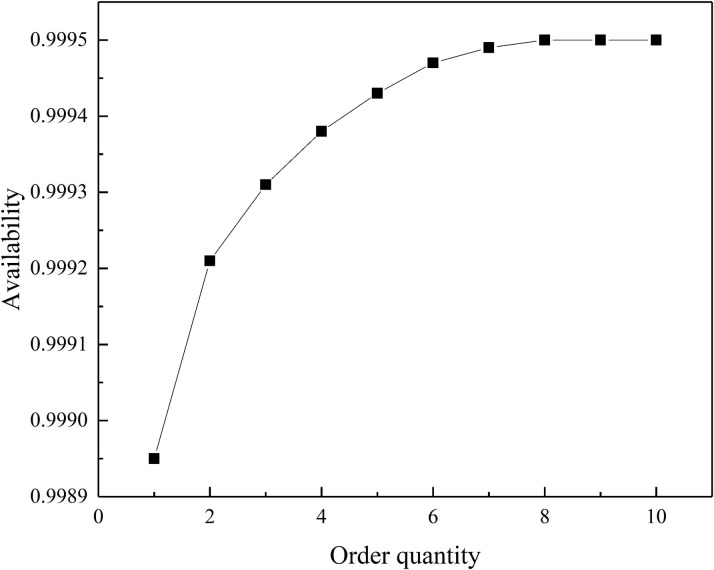
Correlation graph between order quantity and equipment availability.

The analysis of the correlation between order quantity and equipment availability, as shown in Fig 8 below.

This research investigated the impact of order quantity on the availability of overhead contact line insulators. The study found that a fixed order point of 1 resulted in a generally increasing trend in insulator availability as the order quantity went up. However, this growth wasn't linear. When the order quantity was below 6 units, increasing the order quantity led to a rapid rise in insulator availability. However, once the order quantity surpassed 6 units, the additional benefit of larger orders became negligible. This suggests that for scenarios where high insulator availability is crucial, focusing on other factors influencing availability might be more effective. Conversely, if a lower level of insulator availability is acceptable, strategically controlling the order quantity can significantly improve availability while keeping costs lower by minimizing unnecessary stockpiling. In essence, this study highlights the existence of an optimal order quantity that balances insulator availability with cost efficiency.

**Fig 9:**
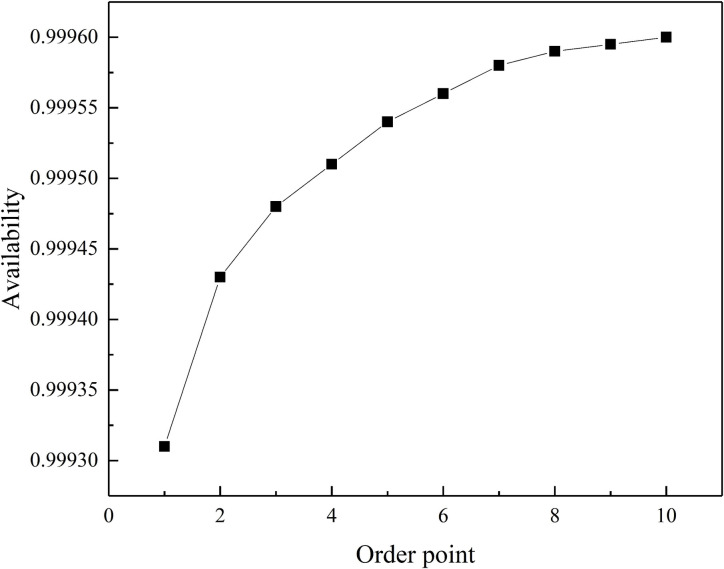
Correlation graph between order point and equipment availability.

The analysis of the correlation between the order point and equipment availability, as shown in Fig 9, is presented below.

When the order quantity is fixed at 3, the downtime due to the lack of spare parts decreases as the order point increases. The availability of overhead contact line insulators consistently increases with the rise in the order point until there is an ample supply of spare parts.

### 5.4. Model sensitivity analysis

By adjusting the main parameters of the model—lead time, fixed procurement cost, unit inventory holding cost per period, unit spare parts shortage cost per period, failure maintenance replacement time, and preventive maintenance replacement time—the impacts on the order point, order quantity, order cycle, equipment availability, total cost per time unit, and spare parts shortage idle time were derived. These results are shown in Table 4.

**Table 4:** Impact of parameter Changes on results.

Case	$T_{L}$ /d	$C_{F}$ /yuan	$c_{s}$ /yuan	$c_{u}$ /yuan	$T_{C}$ /d	${T_{P}}^{\prime }$ /d	r*	Q*	A*	$C_{T}*$ /yuan
1	30	1000	500	2000	0.2	0.2	1	3	0.9993	277020
2	60	1000	500	2000	0.2	0.2	2	3	0.9993	295120
3	90	1000	500	2000	0.2	0.2	2	4	0.9994	306990
4	30	2000	500	2000	0.2	0.2	1	3	0.9993	278020
5	30	1000	1000	2000	0.2	0.2	1	2	0.9992	279860
6	30	1000	500	4000	0.2	0.2	1	3	0.9993	279020
7	30	1000	500	2000	0.3	0.2	2	4	0.9993	281520
8	30	1000	500	2000	0.2	0.3	2	3	0.9993	282350

In Case 1, the optimal strategy for the spare parts inventory model, when the main parameters are set at the values listed in the table, is to order 3 spare parts when the inventory level reaches the order point r* of 1. At this point, the cost is 276,520 yuan, and the availability rate is 99.93%.

Case1–3 demonstrates that an increase in the lead time for orders results in higher order points and quantities, along with an increase in costs. When both the order point and quantity increase significantly, there is a slight improvement in equipment availability.

Comparing Case 1 with Case 4 and Case 5, changes in the fixed procurement cost do not affect the order point and quantity. However, an increase in inventory holding costs reduces the order quantity. This is because, in the scenarios presented, changes in fixed procurement costs have a minor impact compared to changes in inventory holding costs. If the cost per procurement increases significantly, it is advisable to purchase more spare parts at once to spread the procurement costs. Conversely, if inventory holding costs increase significantly, it is preferable to reduce the quantity of spare parts ordered at a time to lower the average inventory level and thus reduce spare parts costs.

Case 6 indicates that the unit time cost of spare parts shortage does not affect the order point, order quantity, or equipment availability, but it does increase the total cost.

Cases 7 and 8 show that an increase in both failure maintenance replacement time and preventive maintenance replacement time leads to higher order points and quantities, with minimal impact on availability.

## 6. Conclusions

This study tackled the challenge of optimizing spare parts inventory for intercity railways, specifically addressing the conflict between component maintenance schedules and the need for adequate spare parts stock. To achieve this, the research integrated two key elements: reliability theory and the established (r, Q) inventory control strategy. A novel ZOA-LSSVM model was developed to accurately model the reliability function of the components. This model then informed the construction of a mathematical inventory control model with the primary objective of minimizing costs. The model considered various constraints, including actual order cycles, order points, order quantities, and required equipment availability. To solve this complex model and achieve optimal spare parts control, a genetic algorithm was specifically designed. The case studies demonstrated the effectiveness of the proposed method. It successfully ensured component availability while maintaining reasonable inventory levels. This translates to preventing excessive stockpiling, minimizing stockout losses, and ultimately reducing overall inventory costs. The proposed method effectively addresses the challenges of spare parts management in intercity railways under normal operating conditions.

The main contributions of this paper are listed as follows. First, the integration of ZOA and LSSVM provides a new method for analyzing the reliability of small sample data spare parts, breaking through the limitations of traditional reliability analysis methods in dealing with scarce data scenarios. The application of this hybrid model fills the gap in the research field of intercity railway spare parts reliability. Second, the theoretical framework of spare parts management for intercity railways has been enriched by combining reliability theory with inventory control strategies, providing a new perspective for future related research. Finally, the established inventory control model and corresponding optimization methods can be directly applied to spare parts management of actual intercity railways, which not only reduces management costs but also improves operational efficiency.

However, this research has several limitations. Firstly, the ZOA-LSSVM model is mainly applied to spare parts with Weibull distribution, and its adaptability to other distribution types of spare parts remains to be further verified. Secondly, the inventory control model established in this study is based on certain assumptions and simplifications of the actual operation environment of intercity railways. In reality, factors such as sudden changes in market prices of spare parts, supply chain disruptions, and policy adjustments may affect the accuracy of the model.

Looking forward, there is potential for further research to explore even more precise methods for fitting reliability functions. Additionally, investigating different inventory control strategies could be beneficial in addressing inventory management issues for intercity railway spare parts under a wider range of scenarios. Specifically, more advanced inventory optimization techniques, such as methods based on reinforcement learning or deep learning, can be introduced and compared with the existing methods. These advanced techniques have shown strong adaptability and optimization capabilities in complex and dynamic environments. By applying and comparing them, we can gain a deeper understanding of their effectiveness and potential in intercity railway spare parts inventory management, and further improve the level of spare parts management.
